# Appropriate use of self-directed learning at medical school prepares students for future clinical practice

**DOI:** 10.1080/10872981.2020.1752450

**Published:** 2020-04-08

**Authors:** Thomas Franchi, Adit Magudia, Adib Rasheed

**Affiliations:** Medical Students, The University of Sheffield Medical School, Sheffield, UK

**Keywords:** Self-directed learning, independent study, integrated learning activities, clinical scenarios, medical education

Dear Editor,

We would firstly like to credit Hill et al. [1] for providing insightful research into the evaluation of self-directed learning for first-year medical students. As fourth-year medical students at The University of Sheffield Medical School, we believe that self-directed learning is an integral aspect of the medical curriculum. As postgraduate examinations are an essential part of career progression, it is paramount for healthcare professionals to be proficient in self-directed learning whilst working in a clinical setting. Therefore, we agree with the sentiments discussed by the authors, and would like to present some additional points with regard to the value of self-directed learning.

The integrated curriculum at our medical school accounts for self-directed learning throughout a students’ progression from the preclinical to clinical years. From the beginning of our medical education, we were introduced to self-directed learning in small groups via Integrated Learning Activities (ILAs). ILAs build on the principles of problem-based learning and are designed to allow students to explore the core clinical situations in collaboration with other medical students that newly qualified doctors often experience. Thus, ILAs help to develop adeptness in communication, problem solving and personal responsibility for learning; all key components of a doctor [2].

Furthermore, our ability to study independently is tested on numerous occasions via written examinations. A significant component of our examinations is formed by the ILAs and a core list of diseases, with a selection of these specifically not taught by the medical school. The examinations, therefore, provide an insight into our ability to carry out self-directed study, as a strong element of independent learning is required by students to progress through medical school.

We feel that an additional point to consider following the contributions made by the Hill et al. is students’ perceived attitudes towards the quality of knowledge obtained as a result of the self-directed learning activity. As medical students, we feel that there is significant difficulty in creating an environment in which we can adequately learn content in-depth under tight time constraints. As a consequence, self-directed study can be undermined, with medical students often lacking confidence in the relevance of information obtained through independent study. This has previously been observed where the preparedness of medical students from different curricula (traditional lecture based versus problem based versus integrated) was assessed. Students in heavily self-directed learning curriculums had a significantly lower self-rating of knowledge compared to students from a traditional curriculum [3]. Therefore, we suggest that medical educators look to address and minimise ambiguity over depth and duration spent on self-learning activities as a way of increasing confidence in the knowledge gained. From our experiences in medical school, we believe there is a distinct difference between students benefiting from self-directed learning versus feeling unsure about whether they have met the intended learning outcomes.

With the ongoing COVID-19 pandemic, and the subsequent temporary but indefinite suspension of the majority of UK medical school teaching and clinical placements [4], it is pertinent that medical students make effective use of their time. By engaging effectively with self-directed learning, students will be able to ensure that they remain informed and competent whilst continuing to develop their knowledge of the standards required to graduate medical school. Whilst learning opportunities will be missed, we believe this can serve as an example to the importance and critical value of self-directed learning in medical education.
